# Nutrition from the kitchen: culinary medicine impacts students’ counseling confidence

**DOI:** 10.1186/s12909-021-02512-2

**Published:** 2021-02-04

**Authors:** Emily Magallanes, Ahana Sen, Milette Siler, Jaclyn Albin

**Affiliations:** 1 UT Southwestern School of Medicine, 5323 Harry Hines Blvd, Dallas, TX 75390 USA; 2 Department of Internal Medicine, UT Southwestern Medical Center, 5323 Harry Hines Blvd, Dallas, TX 75390 USA; 3 Moncrief Cancer Institute at UT Southwestern, 400 W. Magnolia Ave, Fort Worth, TX 76104 USA; 4 Departments of Pediatrics and Internal Medicine, UT Southwestern Medical Center, 5323 Harry Hines Blvd, Dallas, TX 75390 USA

**Keywords:** Culinary medicine, Nutrition education, Medical education, Lifestyle change

## Abstract

**Background:**

Although a poor diet is the number one risk factor for early death in the United States and globally, physicians receive little to no training in dietary interventions and lack confidence counseling patients about lifestyle modifications. Innovative, interprofessional strategies to address these gaps include the emergence of culinary medicine, a hands-on approach to teaching the role of food in health outcomes. We sought to assess the impact of a culinary medicine elective on counseling confidence, awareness of an evidence-based approach to nutrition, and understanding of the role of interprofessional teamwork in dietary lifestyle change among medical students at one undergraduate medical school.

**Methods:**

We administered pre- and post-course surveys to two cohorts of medical students (*n* = 64 at pre-test and *n* = 60 at post-test) participating in a culinary medicine enrichment elective. Chi-square analysis was used to assess the relationship between participation in the course and a positive response to each survey item.

**Results:**

Compared with the baseline, students participating in culinary medicine were more likely to feel confident discussing nutrition with patients (29% vs 92%; *p* < 0.001), to feel familiar with the Mediterranean diet (54% vs. 97%; *p* < 0.001), and to understand the role of dietitians in patient care (37% vs. 93%; *p* < 0.001).

**Conclusions:**

Culinary medicine shows promise as an impactful educational strategy among first-year medical students for increasing counseling confidence, promoting familiarity with evidence-based nutrition interventions, and augmenting understanding of the role of interprofessional engagement to address lifestyle-related disease.

**Supplementary Information:**

The online version contains supplementary material available at 10.1186/s12909-021-02512-2.

## Background

Worldwide, a low quality, nutrient-poor diet is a top risk factor for all-cause mortality and disability [1, 2]. A standard Western diet has been associated with increased risk of heart disease, stroke, type 2 diabetes, cancer, and Alzheimer’s disease [1, 3–5]. Furthermore, obesity and obesity-related chronic disease [6] account for an estimated 75% of the US healthcare budget or $2.6 trillion annually [7]. Despite these overwhelming numbers, estimates show that regular dietary counseling by physicians occurs in only 20 to 40% of patient encounters [8–13]. Physicians cite several factors for their dearth of dietary counseling, including insufficient time and compensation as well as lack of knowledge, training, and self-efficacy to engage in diet-related counseling behaviors [14–19]. This may be due to the well-documented deficit of nutrition training in both undergraduate and graduate medical education [20–34]. Fewer than one quarter of US medical schools provide the recommended number of hours of nutrition education, and schools describe current nutrition education efforts as primarily in a preclinical didactic setting with minimal emphasis on the role of nutrition in clinical practice [20]. Ensuring adequate preventative care for patients is a complex issue that is not easily solved by any institution or program. However, institutions can more feasibly focus efforts on educational innovation to ensure that medical students, residents, and fellows receive adequate nutrition education in order to support the preventative health goals of patients.

There have been many prior efforts to establish effective nutrition education programs in medical schools. Perhaps most notable is the Nutrition Academic Award (NAA) funded by the National Institutes of Health which supported medical schools in the development of enhanced nutrition education programs. Participating schools developed such programs as part of their required undergraduate medical education (UME) curricula and networked with national organizations to promote furtherance of national UME nutrition education standards [35]. An impact evaluation of one NAA curriculum implemented at a single medical school found that participating students had greater nutrition knowledge and self-efficacy compared to controls [36]. Other nutrition and preventive medicine curricula implemented at US medical schools, many of whom also received an NAA, have had similar findings with increases in counseling confidence and knowledge as well as improved self-reports of personal dietary habits [37, 38]. Unfortunately, although nutrition education among medical students is effective, the lasting impact of many prior interventions remains disappointing. Neither the efforts of the NAA nor of independent medical schools have resulted in the creation of widely accepted or implemented standards for curricula, with nutrition education adoption still sparse and heterogeneous [20, 21, 26, 32, 39].

In contrast, a nutrition teaching innovation known as Culinary Medicine (CM) has rapidly gained popularity and prominence among medical schools and community health programs across the US [21]. The most widely adopted CM curriculum, *Health meets Food*, began at Tulane University in 2013 [40]. In 2015, Moncrief Cancer Institute at the University of Texas Southwestern Medical Center was the first to license and launch the *Health meets Food* curriculum. Just five short years later, more than 55 medical schools, residency programs, and nursing schools have adopted *Health meets Food* [41], and many other schools have developed their own programs [42] following a similar model of instruction. The exact reason for the unprecedented success of CM programs is currently unknown but may be due to the unique hands-on teaching model, strong interprofessional collaboration, and a centralized approach that is easy to license and adapt [21, 39].

Unlike nutrition programs based on traditional teaching methods, CM programs teach the role that food plays in health through experiential learning in a kitchen [40, 42]. Interprofessional teams of physicians, dietitians, and chefs combine skills to highlight data-driven strategies that promote delicious, accessible, and healthy cooking. In turn, eating healthy meals serves as a prevention and treatment modality for major chronic diseases. Rooted in a simulation-based medical education approach with deliberate practice (SBME-DP), a model shown to be superior for acquiring and mastering new skills, CM offers learners the opportunity to immediately apply new knowledge with peer support and expert guidance [43]. Guided practice fosters self-efficacy regarding dietary choices, culinary skills, and nutrition counseling as students cook together, eat together, and teach each other about the nutritional value of various foods [44]. While some students may go on to lead culinary demonstrations in their practices or in the community, a growing strategy, all students will gain practical skills that can be applied to discussions with patients about specific dietary changes to improve health. This interactive approach also enables instructors to observe higher level skill competence as defined by Miller’s pyramid [45]. For example, educators can informally assess their students’ knowledge (“knows”) via topic-specific nutrition quizzes, application (“knows how”) via patient case discussions, and demonstration of learning (“shows”) via both live culinary skill demonstration and peer education presentations. This observation of rich educational data happens in real time and on a regular basis.

The *Health meets Food* curriculum has been especially widely adopted through licensing of their convenient and comprehensive courseware. *Health meets Food* espouses the evidence-based Mediterranean diet [46] which emphasizes vegetables, fruits, whole grains, fish, legumes, nuts, and seeds, with olive oil as a primary source of fat [46–53]. The complete courseware currently encompasses over 30 modules on various topics in nutrition. Eight core modules cover foundational principles and additional modules focus on disease or population specific topics such as nutrition and cancer, nutrition in pregnancy, nutrition for heart disease, and mindfulness with motivational interviewing. At Tulane University, all first- and second-year medical students participate in several modules from the core curriculum in addition to elective opportunities. For other institutions utilizing the *Health meets Food* curriculum, the implementation varies both in terms of level of training, timing, and modules selected.

Although CM has been enthusiastically adopted, the reasons for this adoption and the outcomes of such programs on students are still under evaluation. A large prospective, observational cohort study of CM education for medical students at Tulane University demonstrated greater improvement in personal dietary patterns and attitudes about the efficacy of nutrition counseling as compared to traditional clinical education [43]. These results are promising and support the aforementioned premise that any enhanced nutrition education for students is impactful. However, additional research is needed to fully understand the specific potential of the CM approach as a solution to the deficit of medical nutrition education in the US, especially when curricula are implemented outside of the institution of origin through licensure. In this study, we will report how a CM elective course implemented among first-year students at one US medical school unaffiliated with Tulane University affected student counseling confidence, familiarity with evidence-based nutrition interventions, and understanding of the role of interprofessional engagement to address lifestyle-related disease. We will also propose directions for future research to build on the current understanding of CM as a nutrition education strategy that is both educationally effective and widely adopted in a sustainable way.

## Method

### Subjects

We surveyed 64 first-year medical students participating in the CM elective at our medical school using an uncontrolled before-and-after study design. The first cohort of 32 students enrolled during the 2017–18 school year. The second cohort of 32 students enrolled during 2018–19.

All first-year medical students (class size approximately 240 students) at our institution were eligible for participation in this study. No other inclusion or exclusion criteria were defined. Recruitment occurred through informal announcement at an enrichment elective fair, by email, and through a first-year class social media page. Electronic enrollment opened at a pre-advertised time, and the available elective slots filled in under a minute in a first-come, first-served manner.

### Intervention

Prior to this study, there were no cohesive electives or required nutrition, prevention, or lifestyle medicine educational courses available for students at our medical school. Consequently, we designed a CM elective using the *Health meets Food* curriculum to meet student demand for enhanced clinically relevant nutrition education.

We divided the 32 students enrolled annually into two groups of 16 to optimize the hands-on nature of the course for a total of 4 groups and 64 students over 2 years. Each group met monthly in the evening for a 3-h module in an on-campus teaching kitchen and experienced an identical curriculum. We utilized the first eight modules in the *Health meets Food* curriculum to teach this course. These topics encompassed the core CM curriculum for medical students and included 1) Introduction to Culinary Medicine, 2) Weight Management and Portion Control, 3) Fats, 4) Food Allergy and Intolerance, 5) Protein, Amino Acids, Vegetarian Diets, and Eating Disorders, 6) Sodium, Potassium, and Hypertension, 7) Carbohydrates, and 8) The Pediatric Diet.

Participants spent most of class time in the teaching kitchen developing practical skills for making appetizing, nutritious, and economical dishes while learning how to discuss health behaviors with patients. Each class started with a small group discussion about clinical cases related to the module topic and a culinary demonstration to illustrate a relevant food science principle. Students then worked in small groups of 4 to prepare assigned recipes and presented their dishes to the rest of the class, highlighting palatable features of the food as well as nutrition content relevant to the module topic. Students subsequently ate together during a discussion about the nutrition science, medical research, and patient care applications relevant to the module.

A medical doctor (MD) and a registered dietitian (RD) co-facilitated the course and group discussions. We further promoted interprofessional education by engaging medical students (prior culinary medicine program graduates) and dietetic student interns as peer mentors in a variety of modalities. Medical student peer mentors supported grassroots recruitment efforts, organized volunteers for kitchen cleaning, and guided the elective participants in patient case discussions related to the module topic. Dietetic student peer mentors aided the course facilitators in pre-module kitchen set-up and with practical support for elective participants during the live cooking, particularly by helping participants prepare to discuss the nutrition highlights of their food during the peer education portion of class. An average of 3–5 peer mentors supported each of the 32 classes in this study.

### Survey instrument and administration

With a multidisciplinary team of dietitians, physicians, and students, we developed a survey unique to this intervention to assess student eating patterns and perceived awareness about the importance of nutrition in health outcomes before and after taking this course. We scored responses based on a traditional 5-point Likert scale ranging from strongly agree to strongly disagree.

Our pre-course survey (Additional file 1) included questions regarding students’ personal wellness habits, beliefs about nutrition and nutrition counseling, confidence discussing nutrition with patients, and perceived familiarity with the Mediterranean diet and the role of RDs in a patient-care team. The post-course survey (Additional file 2) included additional questions related to students’ satisfaction with the course and the extent to which they incorporated learned habits into their personal routines.

Students completed pre-course surveys during the first class and post-course surveys on the last day of class. Participation was voluntary and did not affect students’ grade or academic standing, and students completed surveys on paper.

### Statistical analysis

Survey data from the 64 participants were compiled into a single data set. We completed chi-square analysis and calculated odds ratios to assess the association between the CM course and a response of “agree” or “strongly agree” to each question on the survey. The Yates correction for continuity was used for contingency tables with any cell having a value of 5 or less.

## Results

Of the 64 first-year medical student participants, all completed the pre-test, and 60 students completed the post-test. Four students over the two-year period were unable to attend the last day of class, and thus they did not complete a post-course survey. While we did not formally collect student-reported demographic data from elective participants, student characteristics reflected the overall class profile and statistics of our institution’s medical students which currently includes a 50/50 mixture of male and female students, 42.6% Asian/Pacific Islander students, 29.6% Caucasian students, 24.8% underrepresented minority students, and diverse backgrounds as evidenced by 11 home countries, 21 home states, and 12% first generation college students.

### Confidence and beliefs about nutrition in patient care

Baseline and post-test confidence, beliefs, and familiarity with the Mediterranean diet and the role of dietitians are summarized in Table 1. At baseline, most students (94%) agreed or strongly agreed that speaking to patients about their food choices is essential. However, only a minority (29%) of students felt comfortable doing so. Few students (37%) understood the role of a dietitian, and just over half (54%) were familiar with the Mediterranean diet.

**Table 1:** Medical student confidence, beliefs, and familiarity with principles of nutrition in patient care

Question	Agree or strongly agree (%)				
	Baseline	Posttest	X^**2**^	***P***-value	OR
Speaking with our patients about their food choices is an essential part of any discussion about health.	94	97	0.11^a^	0.736	1.93
I am comfortable having a discussion with a patient about eating habits and health with my current level of nutrition knowledge.	29	92	47.8^a^	< 0.001	26.8
I am familiar with the basic tenets and research associated with the Mediterranean diet.	54	97	28.43^a^	< 0.001	25.59
I feel confident that I know what a dietitian does and how they might fit into a patient care team.	37	93	39.77^a^	< 0.001	23.3
I believe that a physician’s personal health habits correlate directly with patient outcomes.	66	92	10.07^a^	0.002	5.5

^a^Indicates use of Yates correction for continuity

The post-test assessment demonstrated significant increases in a number of measures. Notably, 92% felt comfortable discussing nutrition with a patient (OR = 26.8), 97% felt familiar with the Mediterranean diet (OR = 25.59), and 93% were confident they understood the role of a dietitian (OR = 23.3).

### Perceptions about personal Health habits

As demonstrated in Table 2, the CM intervention significantly increased the number of students that felt confident in the kitchen (OR = 3.26) and significantly decreased those that believed healthy eating is expensive and time-consuming (OR = 0.43). There was no significant change in the number of students that made food for themselves at home.

**Table 2:** Medical student perceptions about personal health habits

Question	Agree or strongly agree (%)				
	Baseline	Posttest	X^**2**^	***P***-value	OR
I enjoy cooking and feel confident in the kitchen.	73	90	5.62	0.018	3.26
Healthy eating is important, but it is expensive and time-consuming^a^	56	35	5.24	0.022	0.43
Even though I am busy, I make time to prepare healthy food for myself.	52	63	1.75	0.185	1.62

^a^For this item, correlation was measured to “disagree” and “strongly disagree”

### Course satisfaction

Participants were overwhelmingly satisfied with their CM experience. 100% agreed that the information they learned was helpful; 93% planned to implement what they learned into their daily lifestyle, and 98% agreed they would recommend this course to others.

## Discussion

Culinary Medicine education has emerged as one intervention in response to calls for better nutrition education for patients and practitioners. This study is one of the first to evaluate the outcomes of using the *Health meets Food* CM curriculum to teach nutrition to medical students and, to our knowledge, is the first study to do so independently of the institution of curricular origin, Tulane University. The impact of CM on the attitudes and behaviors of participants, especially within patient encounters, needs further study.

CM training for first-year medical student participants at our institution increased awareness of an evidence-based dietary pattern, familiarity with dietitians and their role on interdisciplinary teams, and self-reported confidence to discuss nutrition in patient care settings. These findings are consistent with other studies that observed increases in confidence and self-efficacy among medical students participating in various forms of nutrition education [36, 37]. We also showed that CM can facilitate changes in beliefs and attitudes about nutrition, an important predictor of student and physician likelihood of providing dietary counseling to their patients [16, 27, 34, 54–56]. This builds upon prior findings in the Tulane University study that demonstrated that CM improves medical student attitudes about nutrition counseling even more than other forms of nutrition education [43]. Our study enhances prior findings by demonstrating similar student outcomes through licensing and application of the same curriculum at another medical school.

Compared to traditional methods, CM is a promising approach to nutrition education because students experience nutrition through the lens of practical, real-life eating strategies set in the context of patient care and counseling. While other curricula are didactic-based and often focus on the biochemical aspects of nutrition, CM trains the learner to first attend to his or her own personal health habits so that they are able to speak to patients with personal conviction and experience. Studies reveal that physicians who practice good personal nutrition habits are more effective and more consistent when counseling patients about diet [23, 54–58]. Additionally, CM introduces learners to food-specific motivational interviewing skills which are essential for carrying out patient education but sadly lacking from most other nutrition and medical school curricula. This interactive, person-centered learning approach may be the reason that CM has gained such rapid attention among the medical community and even the popular media [59–61].

An additional strength of using a CM approach to nutrition education is the interdisciplinary perspectives—dietitians, physicians, and chefs—and the opportunity to work hand-in-hand with professionals from these fields. These interactions may have contributed to the observed increase in understanding of the role of dietitians and may pave the way for increased collaboration between physicians and other health and culinary professionals in the future. This interprofessional approach will likely be essential in creating cultural change regarding the emphasis on food as a prevention and wellness strategy.

There are several notable strengths of this study. First, we focused on student beliefs, attitudes, and perceptions of self-efficacy which are known to play a positive role in impacting counseling comfort with patients [62]. Second, our analysis encompasses two consecutive years of data which allowed us to demonstrate the consistent impact of our curriculum repeated for different cohorts of students. The course instructors and curricular topics presented remained unchanged across the 2 years, allowing assessment of very similar course experiences. Finally, while not a measured outcome, the ongoing peer mentor engagement enabled medical students at higher levels of training to have ongoing exposure to the nutrition curriculum through volunteering.

We encountered several limitations. First, we obtained our data from one medical school with courses taught by one set of instructors, thus limiting assumptions about generalizability. However, as previously discussed, our findings aligned with the results of prior work at the curriculum’s founding institution [63]. Finally, any possible differences between the two cohorts, including demographic stratification, were not assessed.

Culinary Medicine is a promising intervention that merits further research. Specifically, future studies are needed to determine the degree to which the positive effects of CM education endure across UME into residency. Additionally, future studies need to assess student behavior in addition to attitudes and should strive to identify ways to assess the impact of CM education on medical student knowledge, interpersonal skills, and motivational interviewing competencies in real or simulated patient encounters.

## Conclusion

In this study, we demonstrate that a CM curriculum can increase awareness of evidence-based nutrition principles and counseling confidence among first-year medical students as well as improve their understanding of interprofessional teams. Additional research is needed to determine if CM increases actual counseling competency and to determine if positive outcomes persist through UME and translate to graduate medical education and physician clinical practice.

## Supplementary Information

**Additional file 1.** Culinary Medicine Elective Pre-Course Survey.

**Additional file 2.** Culinary Medicine Elective Post-Course Survey.

## Data Availability

The datasets used and/or analyzed during the current study are available from the corresponding author on reasonable request.

## Abbreviations

CM: Culinary Medicine
MD: Medical Doctor
RD: Registered Dietitian
UME: Undergraduate Medical Education
