# Supplementary material for: Nutrition from the kitchen: culinary medicine impacts students’ counseling confidence
Source: BMC Med Educ. 2021 Feb 4;21:88. doi: 10.1186/s12909-021-02512-2 (PMC7863372; doi:10.1186/s12909-021-02512-2)
Supplement: Supplementary file 1 — Additional file 1. Culinary Medicine Elective Pre-Course Survey. [file 12909_2021_2512_MOESM1_ESM.docx]

Welcome to Culinary Medicine. We hope that you have a wonderful experience. Please fill out BOTH SIDES of this survey to help us improve the class for you and others.

**Place an X in the box that best represents how you feel about each statement.**

|  | Strongly agree | Agree | Neither agree/ disagree | Disagree | Strongly disagree |
| --- | --- | --- | --- | --- | --- |
| I believe that a physician’s personal health habits correlate directly with patient outcomes. |  |  |  |  |  |
| I enjoy cooking and feel confident in the kitchen. |  |  |  |  |  |
| I am comfortable having discussion with a patient about eating habits and health with my current level of nutrition knowledge. |  |  |  |  |  |
| Even though I am busy, I make time to prepare healthy food for myself. |  |  |  |  |  |
| Healthy eating is important, but it is expensive and time-consuming. |  |  |  |  |  |
| Chronic stress is a part of my day to day life. |  |  |  |  |  |
| I feel like I know how to manage my stress level in healthful ways. |  |  |  |  |  |
| I feel that my academic institution supports me in finding a healthful balance between school and personal time. |  |  |  |  |  |
| I am familiar with the basic tenets and research associated with the Mediterranean Diet. |  |  |  |  |  |
| Speaking with our patients about their food choices is an essential part of any discussion about health. |  |  |  |  |  |
| I feel confident that I know what a dietitian does and how they might fit into a patient care team. |  |  |  |  |  |

**Eating Habits**

1. Usually, I eat _____ servings of **vegetables** daily (one serving of vegetables is ½ cup cooked vegetables or 1 cup of raw vegetables)
2. Usually, I eat _____ servings of **fruits** daily (one serving of fruit is 1 cup of whole fruit such as a small apple, ½ cup of fruit juice or ¼ cup of dried fruit)
3. Usually, I eat _____ servings of **meat** daily (one serving of meat is 3 oz, or the size of a deck of cards)
4. Usually, I eat _____ servings of **whole grains** daily (one serving of grains is one slice of bread, ½ cup of pasta/rice/cereal, 1 cup of cereal). Whole grains include 100% whole grain bread, pasta, brown rice, etc…)
5. Usually, I eat ______ servings of **dairy** daily (one serving of dairy is 1 cup of milk or yogurt, or 1 slice of hard cheese or 1/3 cup of shredded cheese)
6. I consume ___ **alcoholic** drinks per week (1 serving is 5 oz of wine, 12 oz of beer, 1.5 oz of liquor)
7. I consume ____ **sodas** per day (1 serving is 12 oz of soda)

If you consume soda, what type is it?

1. Diet soda
2. Sugar-sweetened soda (regular)

Place an X in the box that applies to you most accurately.

|  | Always | Very Often | Sometimes | Rarely | Never |
| --- | --- | --- | --- | --- | --- |
| 8. I eat breakfast |  |  |  |  |  |
| 9. I skip meals |  |  |  |  |  |

Briefly, please answer the following questions:

1. Have you received formal nutrition or culinary training associated with your schooling? If yes, please describe content and duration:
2. Why did you decide to take this course?
3. What are your goals/desired outcome of this course?
