# Supplementary material for: Nutrition from the kitchen: culinary medicine impacts students’ counseling confidence
Source: BMC Med Educ. 2021 Feb 4;21:88. doi: 10.1186/s12909-021-02512-2 (PMC7863372; doi:10.1186/s12909-021-02512-2)
Supplement: Supplementary file 2 — Additional file 2. Culinary Medicine Elective Post-Course Survey. [file 12909_2021_2512_MOESM2_ESM.docx]

We would like to thank you for your participation in the Culinary Medicine course!

Please take this brief survey to assess the impact of the class and to assist us in making useful improvements. Your constructive comments are welcome.

**Place an X in the box that best represents how you feel about each statement.**

|  | Strongly agree | Agree | Neither agree/ disagree | Disagree | Strongly disagree |
| --- | --- | --- | --- | --- | --- |
| I believe that a physician’s personal health habits correlate directly with patient outcomes. |  |  |  |  |  |
| I enjoy cooking and feel confident in the kitchen. |  |  |  |  |  |
| I am comfortable having discussion with a patient about eating habits and health with my current level of nutrition knowledge. |  |  |  |  |  |
| Even though I am busy, I make time to prepare healthy food for myself. |  |  |  |  |  |
| Healthy eating is important, but it is expensive and time-consuming. |  |  |  |  |  |
| Chronic stress is a part of my day to day life. |  |  |  |  |  |
| I feel like I know how to manage my stress level in healthful ways. |  |  |  |  |  |
| I feel that my academic institution supports me in finding a healthful balance between school and personal time. |  |  |  |  |  |
| I am familiar with the basic tenets and research associated with the Mediterranean Diet. |  |  |  |  |  |
| Speaking with our patients about their food choices is an essential part of any discussion about health. |  |  |  |  |  |
| I feel confident that I know what a dietitian does and how they might fit into a patient care team. |  |  |  |  |  |

Place an X in the box that best represents how you feel about each statement since taking part in the Culinary Medicine program.

|  | Strongly agree | Agree | Neither agree/disagree | Disagree | Strongly disagree |
| --- | --- | --- | --- | --- | --- |
| The information I learned throughout this class was helpful. |  |  |  |  |  |
| I have made changes to my eating habits over the course of this class. |  |  |  |  |  |
| I have made changes to my physical activity over the course of this class. |  |  |  |  |  |
| I plan to implement the information I have learned from this class into my daily lifestyle. |  |  |  |  |  |
| I would recommend this course to my coworkers/patients. |  |  |  |  |  |

Comments:
